# Drug-Eluting Stent Use in Percutaneous Coronary Interventions—A Narrative Review

**DOI:** 10.3390/jcm14134643

**Published:** 2025-07-01

**Authors:** Rok Arh, Igor Balevski, Samo Granda, Sebastjan Bevc

**Affiliations:** 1 Clinical Department of Cardiology, Clinic for Internal Medicine, University Clinical Centre Maribor, 2000 Maribor, Sloveniaigor.balevski@ukc-mb.si (I.B.); samo.granda@ukc-mb.si (S.G.); 2 Department of Nephrology, Clinic for Internal Medicine, University Clinical Centre Maribor, 2000 Maribor, Slovenia; 3 Faculty of Medicine, University of Maribor, 2000 Maribor, Slovenia

**Keywords:** drug-eluting stent, coronary artery disease, percutaneous coronary intervention, taxanes, mammalian target of rapamycin inhibitors

## Abstract

Coronary artery disease is the most common cause of mortality worldwide. Percutaneous coronary intervention represents an important method of treatment. Over time, the methods have been refined to improve safety and efficacy. With the development of drug-eluting stents, in-stent restenosis has importantly decreased, but it remains a relevant concern in terms of the need for additional revascularization procedures or recurrent coronary events. Different platforms, polymers, and anti-proliferative agents have been tested, mostly demonstrating non-inferiority when compared. Additional devices, such as drug-coated balloons, bioresorbable scaffold systems, gene-eluting stents and bioadaptor implants have been developed. As none of the aforementioned methods demonstrated considerable superiority over the others, the search for the ideal treatment method continues. Based on currently available data, the ideal treatment method could be a personalized approach combining different revascularization methods. Additional research with subpopulation group studies, different associated diseases or vessels affected, and longer follow-up are required to determine better subgroups of patients that would benefit most from specific treatment methods.

## 1. Introduction

### 1.1. Objectives of the Review

This narrative review aims to provide a comprehensive and up-to-date overview of the development and outcomes associated with different drug-eluting stents (DESs) in percutaneous coronary interventions (PCIs). Specifically, the objectives of this narrative review were to summarize the evolution of DES technology, compare the characteristics, advantages, and limitations of different generations and types of DESs used in clinical practice, emphasize key findings from major clinical trials and studies evaluating the efficacy and safety of DESs, and explore current innovations in DES design.

### 1.2. Methodology

A literature search was conducted using the PubMed database for this narrative review. The search used the keywords: drug-eluting stent, percutaneous coronary intervention, and outcome comparison. All relevant articles published in English up to the time of writing were considered, without applying specific date restrictions. Articles were selected based on their focus on the clinical use, comparative outcomes, advancements, and safety profiles of drug-eluting stents in the context of percutaneous coronary interventions. Additional references were identified through a manual screening of citations within key articles to ensure a comprehensive overview of the topic.

### 1.3. Coronary Artery Disease

Coronary artery disease (CAD) represents the most common single cause of mortality. Overall, it is reported to be a cause of approximately 20% of all deaths in the developed world. In the United States, CAD has been shown to affect 16.8% of people. The disease is characterized by the atherosclerosis of coronary arteries or non-atherosclerotic condition resulting in ischemic heart disease; either acute coronary syndrome (ACS—unstable angina pectoris, non-ST elevation myocardial infarction (NSTEMI), and myocardial infarction with ST elevation (STEMI)) or chronic coronary disease, which can manifest in stable angina pectoris or silent myocardial ischemia [1,2].

### 1.4. Percutaneous Coronary Intervention

PCI represents the gold standard procedure for the treatment of the acute presentation of CAD. Fibrinolysis is mainly used in settings where PCI is not available in a timely manner. Additionally, coronary artery bypass grafting (CABG) can be used in complex CAD—multivessel disease or sometimes left main coronary artery disease [2,3]. In chronic coronary artery disease, either optimal medical therapy or PCI or CABG revascularization can be used based on individual patient assessment [4]. Approximately 1400 PCI procedures per million are performed yearly in the United Kingdom [5].

### 1.5. Evolution of Stents

Coronary angioplasty was conceptually described by Dotter and Judkins in 1964 and was first performed in 1977 by Gruntzig. The first angioplasty procedures were performed without stent deployment as plain old balloon angioplasty (POBA), and stents were introduced in the mid-1980s. With their use in clinical practice, coronary artery dissection and vascular recoil were reduced because of the expandable, metallic meshwork of the stent. The first bare metal stent (BMS) approved for acute closure was approved in 1993, followed by the first bare metal stent approved for elective use in 1994 [5,6,7]. It is reported that using BMSs reduced the incidence of restenosis after angioplasty to 20–53% [8,9].

As immediate vessel recoil after stretch injury and negative arterial remodeling were importantly reduced using BMSs, the biggest obstacle to overcome was in-stent restenosis (ISR), which can arise from multiple mechanisms including neointimal hyperplasia, stent underexpansion, stent fracture, undersizing, neoatherosclerosis, and late stent recoil, and was reported to occur in 15–30% [7,8]. In ISR pathophysiology, smooth muscle cell activation and replication occur at the site of injury; thus, the idea for resolving the problem was to locally deliver an appropriate concentration of an effective agent to stop this process without systemic toxicity [7]. To overcome this problem DESs were invented. Their use has shown an important improvement considering ISR, as it was reduced to less than 10% in clinical trials, with the need of additional revascularization procedures importantly reduced. The studies showed their superiority over BMSs even in more complex lesions and acute coronary events [8,10,11,12,13,14,15].

First-generation DESs consist of a stainless-steel base with either a sirolimus or paclitaxel coating. Second-generation DESs normally use a biocompatible cobalt–chromium, platinum–chromium, or nickel–titanium platform coated with zotarolimus or everolimus. Biolimus, a sirolimus derivate with increased lipophilicity, is also used. Pores in the polymeric coating allow for diffusion of the therapeutic agent [6,9,16,17].

Although the latter has offered good efficacy and safety, the search for a better antiproliferative agent continues. Different newer agents are being tested including novolimus, a metabolite of sirolimus, and myolimus [5].

To reduce the hypothetical pathophysiological mechanism of hypersensitivity reaction in durable polymer stents of the first- and the second-generation, a third-generation with bioresorbable polymer coating was created [17].

Most current PCI procedures include stent deployment following prior balloon dilatation of the affected vessel [5]. Afterward, for a longer duration of dual anti-platelet therapy, this was overcome by the current-generation DESs. Current-generation DESs can overcome the need for a longer duration of dual anti-platelet therapy with reduced strut thickness and better or no polymers [5,9].

In addition to DESs, drug-coated balloons (DCBs) and bioresorbable scaffold systems (BRSs) were invented. DCBs consist of a balloon, covered with an antiproliferative agent without an underlying metal structure of a stent. Studies have shown promising results of its use, especially in small coronary artery disease [6,18,19,20]. Some studies have also reported non-inferiority in large coronary artery disease [21,22]. BRSs are also similar to stents lacking a metallic structure. It is completely reabsorbed after a few months, after serving its purpose [6]. Meta-analysis and systematic review focusing on a BRS and DES comparison showed an increased risk of target lesion failure within 1 year in the BRS group compared with the DES group [23].

## 2. Stent Structure

DESs consist of three components: a metallic platform, a therapeutic agent that prevents neointimal growth, and a drug carrier vehicle that stores that therapeutic agent and allows it to diffuse into local tissue in a controlled fashion [6,7].

As above-mentioned, different materials and drugs can be used on either level of stent structure [6,7,9].

### 2.1. Metallic Platform

Most of the first-generation stents were made of stainless-steel, as it provides adequate radial strength to restore the patency of a stenotic artery. Although it is sometimes still used in currently used stents, alloys are used more often. These include cobalt–chromium, platinum–chromium, and nickel–titanium. Some stents also consist of two alloys, an example being an outer shell of a cobalt-based alloy with a platinum–iridium core to increase radiopacity. In comparison with stainless-steel, these alloys have an intrinsically greater tensile strength whilst possessing similar or reduced elasticity, which allows for the manufacturing of thinner struts (typically >100 μm for stainless-steel versus <100 μm for alloys) whilst maintaining strength, improving the flexibility and deliverability, and reducing the risk of stent thrombosis (ST). It is reported that the strut thickness and stent flexibility have an impact on the degree of injury, risk of rupture of the elastic laminae, and overall inflammation, which may increase the risk of ST after implantation. In addition, first-generation DES studies have shown that thicker struts were associated with a higher incidence of side branch occlusion compared with thinner-strut DESs. Thinner-strut devices have also been associated with better clinical outcomes [9,24,25].

### 2.2. Polymers

The drug carrier vehicle used in first-generation non-degradable primarily stents was mostly non-degradable synthetic polymers such as polyethene-co-vinyl acetate, poly-n-butyl methacrylate, and the tri-block copolymer poly(styrene-b-isobutylene-b-styrene). As histopathological data indicated that the delayed vascular healing due to these polymer coatings may be associated with an increased risk of very late ST after first-generation DES implantation, more biocompatible permanent polymers such as phosphorylcholine, a co-polymer of poly-vinylidene fluoride and hexafluoropropylene were developed. Further development of DESs has led to biodegradable polymers; these behave as conventional DESs in the early phase and revert to BMSs when all the drug has been released. These typically utilize polylactic acid or a variation as the polymer, which is degraded by hydrolysis over months to lactic acid. The last to have been introduced to the practice were polymer-free DESs. The drug is directly applied to the metallic surface without utilizing a polymer, which has the theoretical advantage of avoiding polymer-related complications [5,9].

### 2.3. Anti-Proliferative Agents

A vast variety of anti-proliferative agents have been tested to date. Mammalian target of rapamycin inhibitors have prevailed. Currently, the agents most often used are sirolimus, everolimus, zotarolimus, or biolimus. In the first-generation DESs, paclitaxel was also used. The search for an ideal anti-proliferative agent used in DESs continues [6,9].

#### Mechanism of Action

**Figure 1:**
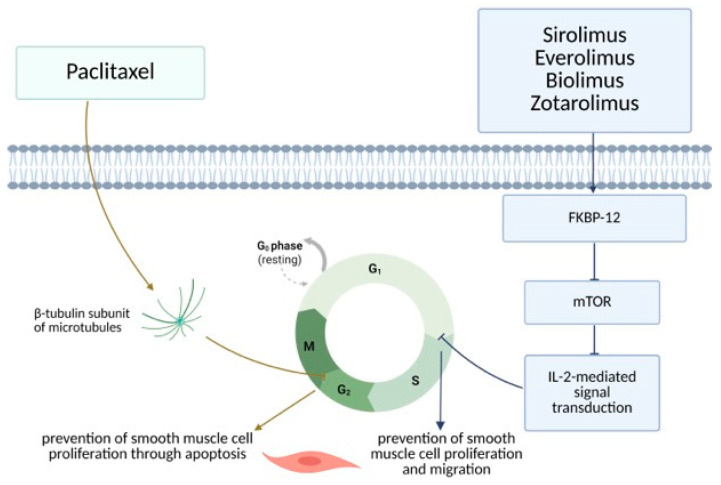
DES—mechanism of action (Created in BioRender. Arh, R. (2025) https://BioRender.com/mh4cnwv, accessed on 24 June 2025).

Currently used anti-proliferative agents are divided into two main groups: rapamycin agents (sirolimus and its derivatives everolimus, zotarolimus, and biolimus) and taxanes (paclitaxel). The mechanism of action of both groups is similar to some extent, as they both block signal transduction and therefore interfere with the cell cycle at different stages. In such a manner, they prevent vascular smooth muscle cell proliferation and intimal hyperplasia at the site of stent implantation. Rapamycin and its derivatives bind to FKBP-12, an intracellular protein that inhibits the protein kinase mammalian target of rapamycin (mTOR). Therefore, it blocks IL-2 (interleukin-2) mediated signal transduction, leading to cell cycle blockade from the G1 phase to the S phase in DNA (deoxyribonucleic acid) synthesis. On the other hand, taxanes directly bind to the β-tubulin subunit of microtubules, and, in that way, interfere with the microtubule function and prevent the M phase of mitosis. In this way, cells are halted in the G2 phase of the cell cycle, which leads to apoptosis [6,26,27] (Figure 1).

Neointimal hyperplasia suppression peaks during the first 4–12 weeks after implantation, aligning with the release period of most DESs, as reported in the literature. Clinical correlated with maintaining drug concentrations above the inhibitory threshold for 28 days or longer. A shorter duration was reported to show a higher ISR rate [28,29,30]. For sirolimus, the stent release time was reported to be 28 days with a tissue half-life of 9 days and functional activity of 3 months [30]. The stent release time for everolimus was reported to be 120 days, with a tissue half-life of 14 days and functional activity of 4 months [17]. For biolimus, approximately 80% of the agent was released from the stent in 30 days. Specific tissue half-life was not measured, but high tissue-uptake and retention were reported due to 10 times higher lipophilicity compared with sirolimus. Functional activity was reported to last longer than 9 months post-implantation [31,32,33,34,35]. A total of 50% of zotarolimus was reported to be released at 7 days and 85% at 60 days. The tissue half-life was not specifically quantified, but the functional activity was reported to be comparable to everolimus [36,37]. In the case of paclitaxel, a stent release time of 28 days was reported with a tissue half-life of 7–10 days and functional activity of 6–8 weeks [29].

## 3. Drug-Eluting Stent Comparison

Studies have compared different stent generations with different stent platforms, therapeutic agents carried out in different platforms, and therapeutic agents carried out in different settings [38].

A study comparing five different DES types (cobalt–chromium everolimus-eluting stents, platinum–chromium everolimus-eluting stents, Resolute zotarolimus-eluting stents, biodegradable polymer biolimus-eluting stents, and first-generation sirolimus-eluting stents) in long lesions comparing in-segment late lumen loss at 9 months was performed, which showed no significant difference between the aforementioned stents [38].

The IRIS-DES study compared five different stent types: durable polymer cobalt–chromium everolimus-eluting stents, durable polymer Resolute zotarolimus-eluting stents, ultrathin strut biodegradable polymer platinum–chromium everolimus-eluting stents, ultrathin strut biodegradable polymer cobalt–chromium sirolimus-eluting stents, and bioresorbable polymer sirolimus-eluting stents. In the study, it was reported that at 12 months, the observed incidences of target-vessel failure were highest in the durable polymer cobalt–chromium everolimus-eluting stent group (7.1%) and lowest in the ultrathin strut biodegradable polymer cobalt–chromium sirolimus-eluting stent group (3.8%). In the other groups, its occurrence was intermediate (in the durable polymer Resolute zotarolimus-eluting stent group 5.0%, ultrathin strut biodegradable polymer platinum–chromium everolimus-eluting stent group 4.6%, and bioresorbable polymer sirolimus-eluting stent group 4.2%). However, as periprocedural enzymatic myocardial infarction without documented ischemia was excluded, the difference between the groups was less prominent. In the analysis, which included only non-procedural myocardial infarction, the overall rates of target-vessel failure were not significantly different [39]. The IRIS-DES registry has additionally been used to assess target vessel failure in patients with diabetes mellitus at 3 years after PCI comparing treatments with a cobalt–chromium everolimus-eluting stent, a biodegradable polymer biolimus-eluting stent, a platinum–chromium everolimus-eluting stent, and a Resolute zotarolimus-eluting stent. No statistically significant difference between the groups was found [40].

A comparison of paclitaxel-, sirolimus-, and everolimus-eluting stents in left main coronary artery PCI in patients with an increased risk of adverse surgical outcomes was performed, showing no statistically significant difference in major adverse cardiovascular events, cardiac death, myocardial infarction, target lesion revascularization, and ST among the three groups 30 days and 1 year after the procedure [41].

### 3.1. Stent Platform Comparison

The COMPARE-II and the NEXT trial both compared stainless-steel biolimus-eluting stents with cobalt–chromium everolimus-eluting stents and platinum–chromium everolimus-eluting stents, showing no significant differences in the primary composite endpoint of safety at 12 months or ST rate after 5 years [9,42,43,44].

Two studies (SORT-OUT VIII and EVERBIO-II) compared stainless-steel stents with reference groups. The first one compared stainless-steel biolimus-eluting stents with platinum–chromium everolimus-eluting stents and proved its non-inferiority considering target lesion failure. The latter compared stainless-steel biolimus-eluting stents with platinum–chromium everolimus-eluting stents and showed no significant difference in lumen loss at 9 months [9,45].

The PLATINUM study compared platinum–chromium everolimus-eluting stents against cobalt–chromium everolimus-eluting stents. The difference in target lesion failure and ST rate was not statistically significant. Furthermore, the PLATINUM PLUS trial showed non-inferiority of platinum–chromium everolimus-eluting stents compared with cobalt–chromium everolimus-eluting stents. There were no significant differences in the rates of cardiac death, myocardial infarction, or ischemia-driven target vessel revascularization. The rates of definite or probable ST were comparable between both platforms [9,46,47].

Meta-analyses have demonstrated a significantly lower incidence of ST and myocardial infarction in stents with ultrathin struts compared with those with thick struts. Based on that data, it is therefore implied that stents with thinner struts (alloys) could be better than those with thicker struts (stainless-steel), but that within current-generation DESs, the choice of material itself does not appear to be a factor that affects the clinical outcomes [9,48,49].

### 3.2. Polymer Comparison

Multiple studies have compared stents with a permanent polymer coating to those with a biodegradable coating (BIOFLOW-V, BIONYX, BIO-RESORT, BIOSCIENCE, CENTURY II, COMPARE-II, LEADERS, NEXT, SORT-OUT V, and SORT-OUT VI). These have mainly demonstrated the non-inferiority of different polymer types, rarely proving the superiority of a stent type. The BIOFLOW-V trial compared a cobalt–chromium biodegradable polymer sirolimus-eluting stent against a cobalt–chromium permanent polymer everolimus-eluting stent, illustrating the superiority of the first one in terms of less target vessel failure at 12 months because of a rate of target vessel myocardial infarction. On the other hand, a direct comparison of two platinum–chromium everolimus-eluting stents with either a biodegradable polymer or permanent polymer in the EVOLVE-II trial showed their non-inferiority, but not superiority. The SORT-OUT V trial compared a stainless-steel biolimus-eluting stent with a biodegradable polymer with a first-generation stainless-steel sirolimus-eluting stent with a permanent polymer but failed to prove its non-inferiority in safety and efficiency. However, that was not the case in the other studies that compared stainless-steel biolimus-eluting stents with biodegradable polymer versus non-stainless-steel everolimus-eluting stents, which proved their non-inferiority. The SORT-OUT VI trial compared a biodegradable polymer biolimus-eluting stent with a permanent polymer cobalt–chromium zotarolimus-eluting stent and permanent polymer stainless-steel sirolimus-eluting stent and implied nominal, but not statistically significant superiority of the first [9,42,43,50,51,52,53,54,55,56].

Meta-analyses showed no statistically significant difference in clinical outcomes between the biodegradable polymer stents and permanent polymer ones, even in unstable coronary artery disease [9,57,58,59,60,61]. The TWILIGHT-SYNERGY trial demonstrated equivalent data [62]. Similarly, the DESSOLVE-III trial showed the non-inferiority of bioabsorbable polymer sirolimus-eluting in comparison with the durable polymer everolimus-eluting stent [63]. Even at 10 years follow-up, no significant difference was noted [64].

Not many studies have compared stents within the biodegradable polymer group, one of them being the SORT-OUT VII trial. There was no statistically significant difference between the stainless-steel biolimus-eluting stents and biodegradable polymer cobalt–chromium sirolimus-eluting stents, except for the definite ST rate being higher in the latter. Similarly, the SORT-OUT VIII study showed no statistically significant difference between biodegradable polymer platinum–chromium everolimus-eluting stents and biodegradable polymer biolimus-eluting stents, only a numerical advantage of the first group [9,65,66].

Even fewer studies have been performed comparing polymer-free stents. Polymer-free cobalt–chromium sirolimus-eluting stents have shown less in-stent late lumen loss at 6 months in comparison to permanent polymer stainless-steel paclitaxel-eluting stents, but it is also important to note the strut differences in both groups. Compared with bare metal stents, the stainless-steel polymer-free biolimus-eluting stents demonstrated superior primary safety (cardiac death, myocardial infarction, or ST) and efficacy (target lesion revascularization) in high bleeding risk patients. No difference in rates of definite ST between the groups was noted [9,43,67].

Scarce data are available on head-to-head polymer-free stent comparisons, but the data comparing stainless-steel biolimus-eluting stents and cobalt–chromium sirolimus-eluting stents have shown no statistically significant differences [9,68].

### 3.3. Anti-Proliferative Drug Comparison

Several randomized controlled studies have compared stents eluting different drugs [9] (Table 1).

#### 3.3.1. Biolimus- Versus Everolimus-Eluting Stents

The COMPARE-II trial and the BASKET PROVE II study both compared biolimus-eluting stents with everolimus-eluting stents. The first compared biodegradable polymer biolimus-eluting stents with thin struts with everolimus-eluting stents with a durable biocompatible polymer, proving their non-inferiority. The BASKET PROVE II study compared biodegradable polymer biolimus-eluting DES, permanent polymer everolimus-eluting DES, and thin strut silicon-carbide-coated BMS. Non-inferiority of the biolimus-eluting biodegradable polymer stent was demonstrated in comparison to the everolimus-eluting permanent polymer stent, as superiority in comparison with the thin strut silicon-carbide-coated BMS was manifested [42,69].

#### 3.3.2. Biolimus- Versus Sirolimus-Eluting Stents

The NEXT, SORT-OUT V, and SORT-OUT VII studies compared biolimus- and sirolimus-eluting stents. The NEXT study showed non-inferiority of the clinical and angiographic outcomes comparing both DES types. The SORT-OUT V study compared biodegradable polymer biolimus-eluting stents versus permanent polymer sirolimus-eluting stents, in which the first ones failed to show a convincing improvement in clinical results. In the SORT-OUT VII trial, thin-strut sirolimus-eluting stents demonstrated non-inferiority in comparison with biolimus-eluting stents in unselected patients for target lesion failure at 1 year [43,44,54,65].

#### 3.3.3. Biolimus- Versus Zotarolimus-Eluting Stents

In the SORT-OUT VI study, biodegradable polymer biolimus-eluting stents were compared with permanent polymer zotarolimus-eluting stents. At 3-year follow-up, the clinical outcomes were similar, with no significant difference in safety and efficacy [55].

#### 3.3.4. Everolimus- Versus Sirolimus-Eluting Stents

Everolimus- and sirolimus-eluting stents were compared in the BIOSCIENCE and the CENTURY II studies. In the BIOSCIENCE study, biodegradable polymer sirolimus-eluting stents were non-inferior to durable polymer everolimus-eluting stents for the combined safety and efficacy outcome target lesion failure at 12-month follow-up. Similarly, in the CENTURY II trial, bioresorbable polymer sirolimus-eluting stents showed safety and efficacy profiles comparable to those of permanent polymer everolimus-eluting stents at 9-month follow-up [53,70]. The ABILITY study also compared biodegradable polymer sirolimus-eluting stents with permanent polymer everolimus-eluting stents in patients with diabetes mellitus. Neointimal hyperplasia was measured with optical computed tomography (OCT) at 9–12 months follow-up, showing no statistically significant difference between both groups [71]. The BIOHEART-II study focused on comparing a sirolimus-eluting bioresorbable stent to a cobalt–chromium everolimus-eluting stent, demonstrating non-inferiority in in-stent luminal loss 1 year after PCI and target lesion failure up to 3 years after PCI [72]. In the TARGET All Comers Trial, a group of patients treated with biodegradable polymer sirolimus-eluting stents demonstrated comparable results in safety and efficacy in both acute and chronic coronary syndrome to those treated with permanent polymer everolimus-eluting stents [73].

#### 3.3.5. Everolimus Versus Zotarolimus-Eluting Stents

The DUTCH PEERS and the RESOLUTE trials compared everolimus-eluting stents with zotarolimus-eluting stents. In the DUTCH PEERS study, both stent types showed similar efficacy and safety, and in the RESOLUTE study, the safety and efficacy were comparable at 5-year follow-up [37,74]. A meta-analysis was conducted based on data on zotarolimus- and everolimus-eluting stents, showing no significant differences in acute, subacute, and late definite or probable ST between the two groups [76].

#### 3.3.6. Sirolimus- Versus Zotarolimus-Eluting Stents

The SORT-OUT III study compared sirolimus- and zotarolimus-eluting stents, and the first group demonstrated superiority in terms of major cardiac adverse events at 9-month and 18-month follow-up. The difference in mortality rate at 9 months was not statistically significant, but at 18-month follow-up, the difference in mortality in both groups became statistically significant [75].

Based on the research conducted so far, it would be difficult to claim that the individual differences demonstrated between stents resulted from the use of different drugs, as other characteristics were also present among the stents studied [9].

## 4. Gene-Eluting Stents

Anti-proliferative agents used in DESs also prevent the healing of vascular endothelium and can therefore cause subacute or late ST. To target this, the idea of a gene-eluting stent (GES) has been proposed, where the agent, used to prevent neointimal hyperplasia, is replaced by genes using different mechanisms. Reduced neointimal hyperplasia, the acceleration of re-endothelization, inhibition of thrombosis, and a reduction in inflammation were targeted. Both viral (retroviruses, adenoviruses, adeno-like viruses, lentiviruses) and non-viral vectors (naked plasmid DNA, lipid nanoparticle-based gene delivery, polymeric nanoparticle-based gene delivery) were tested. An ideal vector would be cell specific, exceptionally potent to achieve high transfection/transduction of the target cells, biocompatible with low toxicity and immunogenicity, and highly efficient to allow for rapid uptake and incorporation, high retention, and prolonged expression. Although the preclinical results and results in animal models are promising, the results in clinical trials have not shown the same success [8,77,78].

## 5. Ongoing Development and Future Directions

Different novel methods, stent matrix materials, and drugs are being tested on preclinical or clinical levels [78].

The SORT-OUT X study compared a sirolimus-eluting stent with a newer dual-therapy sirolimus-eluting and CD34 antibody-coated stent, used to improve early healing of the lesion, but target lesion failure in the first year after the PCI was significantly higher in the group receiving a dual-therapy stent, which was also reported in a systemic review and meta-analysis on an endothelial progenitor cell-capturing DES that contained a CD34-antibody coating. The results of target lesion failure after 1 year and up to 3 years after the PCI became almost identical [79,80]. In the SORT-OUT XI trial, a biodegradable polymer biolimus-eluting stent was compared with a biodegradable polymer sirolimus-eluting and CD34+ antibody-coated stent in terms of non-inferiority [16].

Heparin-covered stents have been tested in porcine models but have failed to demonstrate benefit in clinical practice. Platelet glycoprotein IIb/IIIa receptor blocker-coated stents showed the inhibition of platelet thrombi and restenosis in a porcine model that were associated with a lower inflammation rate. They significantly reduced the in-stent neointimal hyperplasia in human coronary arteries, with potential therapeutic benefit in preventing stent restenosis. Therefore, the idea of two-lumen stents—abluminal for anti-proliferative agent and luminal for agent that promotes re-endothelization—was developed [78].

Several antioxidants (carvedilol, probucol, and alpha-lipoic acid) have been tested as stent coatings. In the porcine model, carvedilol was shown to be more effective than probucol. No ST was observed in models with carvedilol stents. Alpha-lipoic acid demonstrated promising in vitro results for patients with diabetes mellitus [78].

Non-polymer coating technology was developed to prevent ST. Polymer-free titanium dioxide stents with abciximab or alpha-lipoic acid were compared with a biolimus-eluting stent in a porcine model, and showed no difference in area of stenosis but accomplished lower fibrin and inflammation levels. A polymer-free DES coated with everolimus using nitrogen-doped titanium dioxide was compared to currently available generation stents in in vitro and porcine models, and the two demonstrated comparable stenosis levels with lower inflammation and fibrin levels in the first group [78].

Dextran-based stents have also been tested, showing enhanced re-reendothelialization and reduced inflammation [78].

Consecutive coating of a BMS with a protein called WKYMV, and later sirolimus, showed the potential to affect re-endothelization and neointimal suppression [78].

Natural binding techniques, including fucoidan, dopamine and phytoncide, have been shown to have a positive impact on either the area of stenosis or the neointimal area, inflammation, and fibrin scores [78].

3D printing systems have allowed for the development of bioabsorbable vascular stents (BVSs). A polycaprolactone stent coated with sirolimus mixed with poly lactic-co-glycolic acid and polyethylene glycol via a spraying method for slow drug release was tested in animal models and reported to lower neointimal hyperplasia [78]. The present meta-analysis demonstrated poorer outcomes than DESs, as an increased rate of recurrent cardiovascular events was reported. However, there was no increase in mortality [81].

Newer devices, bioadaptor implants that restore the hemodynamic modulation of the artery, allow for cyclic pulsatility, vasomotion, and adaptive remodeling by unlocking and providing dynamic support to the artery, have been tested in a single-blind, non-inferiority, registry-based, randomized controlled trial (INFINITY-SWEDEHEART), which showed its non-inferiority compared with contemporary DESs at 1 year follow-up [82].

## 6. Conclusions

CAD remains an important medical problem. Development of DESs and consequent refinement have significantly improved PCI efficacy and safety. As most of the current DES comparisons have demonstrated their non-inferiority in comparison to each other based on polymer and anti-proliferative drugs and late ISR remaining an important challenge, the search for an ideal DES composition regarding its platform, polymer, and drug used continues. It is important to note that most trials were conducted before the widespread use of intracoronary imaging techniques. With the increased use of intravascular imaging, a significant decline in the incidence of adverse events observed in those trials has been detected, making it increasingly challenging to demonstrate differences between different DES platforms. Newer devices like DCBs and bioadaptor implants have been developed, and based on the current data, a personalized approach combining different revascularization methods could prevail. Additional research with subpopulation group studies, different associated diseases or vessels affected, and longer follow-up are needed to better assess subgroups of patients that would benefit the most from specific treatment methods.

## Figures and Tables

**Table 1:** Studies comparing DESs.

Name of the Study	DES Compared	Population	Endpoints Observed	Outcomes	Reference
COMPARE-II	Biodegradable polymer biolimus-eluting stent vs. thin strut durable biocompatible polymer everolimus-eluting stent	2707 patients (age > 18 years, life expectancy > 5 years, reference vessel diameter 2–4 mm)	Cardiac death and non-fatal myocardial infarction or clinically indicated target vessel revascularization at 12 months	93 patients (5.2%) vs. 44 patients (4.8%); p_non-inferiority_ < 0.001	[42]
BASKET PROVE II	Biodegradable polymer biolimus-eluting DES vs. permanent polymer everolimus-eluting DES (additionally vs. thin strut silicon-carbide-coated BMS)	2291 patients presenting with acute or stable coronary disease needing stents ≥ 3.0 mm in diameter	Combined cardiac death, myocardial infarction, and clinically indicated target-vessel revascularization within 2 years	7.6% vs. 6.8%; p_non-inferiority_ = 0.042 12.7% in the BMS group	[69]
NEXT	Biodegradable polymer biolimus-eluting stent vs. durable polymer everolimus-eluting stent	3235 patients who underwent PCI using DES	Death or myocardial infarction	159 patients (9.9%) vs. 166 patients (10.3%); p_non-inferiority_ < 0.001 No significant difference between groups in target-lesion revascularization and cumulative incidence of death or myocardial infarction at 1 year between the 2 groups	[44]
SORT-OUT V	Biodegradable polymer biolimus-eluting stent vs. durable polymer sirolimus-eluting stent	2468 patients aged 18 years or older with chronic stable coronary artery disease or acute coronary syndromes, and at least one coronary artery lesion (>50% diameter stenosis)	A composite of safety (cardiac death, myocardial infarction, definite ST) and efficacy—target vessel revascularization at 9 months	Intention-to-treat analysis, primary endpoint: 50 patients (4.1%) vs. 39 patients (3.1%); p_non-inferiority_ = 0.06 Definite ST at 12 months: 0.7% vs. 0.2%; *p* = 0.034 Per-protocol analysis, primary endpoint: 3.8% vs. 3.2%; p_non-inferiority_ = 0.03	[54]
SORT-OUT VII	The thin strut cobalt–chromium sirolimus-eluting stent vs. stainless-steel biolimus-eluting stent	2525 patients aged 18 years or older, chronic stable coronary artery disease or acute coronary syndrome, and at least 1 coronary lesion with >50% diameter stenosis, requiring treatment with a DES	Target lesion failure—a composite of cardiac death, myocardial infarction (not related to the index lesion), or target lesion revascularization within 1 year	48 patients (3.8%) vs. 58 patients (4.6%); p_non-inferiority_ < 0.001	[65]
SORT-OUT VI	Biocompatible durable-polymer zotarolimus-eluting stent vs. biodegradable-polymer biolimus-eluting stent	2999 patients with chronic stable coronary artery disease or acute coronary syndrome and at least 1 coronary artery lesion requiring treatment with a DES	Major adverse cardiac events; a composite of safety (cardiac death and myocardial infarction not clearly attributable to a non-target lesion) and target lesion revascularization: all-cause mortality; any myocardial infarction; target vessel revascularization; and definite or probable ST at 36 months	Cardiac death: 2.7% vs. 3.4% (not statistically significant) myocardial infarction not clearly attributable to a non-target lesion: 2.7% vs. 2.5% (not statistically significant) Target lesion revascularization: 5.4% vs. 5.5% (not statistically significant) Definite very late ST: 6 patients (0.4%) vs. 10 patients (0.7%); *p* = 0.33	[55]
BIOSCIENCE	Ultrathin strut biodegradable polymer cobalt–chromium sirolimus-eluting stent vs. thin strut durable polymer everolimus-eluting stent	2119 patients aged 18 years or older with chronic stable coronary artery disease or acute coronary syndromes undergoing PCI	Target lesion failure—a composite of cardiac death, target vessel myocardial infarction, and clinically indicated target lesion revascularization at 12 months	Target lesion failure in 69 patients (6.5%) vs. 70 (6.6%) at 12 months; p_non-inferiority_ < 0.001 No significant differences in rates of definite ST 9 (0.9%) vs. 4 (0.4%); *p* = 0.16	[53]
CENTURY II	Bioresorbable polymer sirolimus-eluting stent vs. permanent polymer everolimus-eluting stent	1123 patients requiring PCI with implantation of DES	Absence of target lesion failure at 9 months (composite of cardiac death, target-vessel-related myocardial infarction and target lesion revascularization	95.6% vs. 95.1%; p_non-inferiority_ < 0.001 Cardiac death and myocardial infarction rate were 2.9% and 3.8%; *p* = 0.40 Target vessel revascularization was 4.5% vs. 4.2%; *p* = 0.77 ST rate was 0.9% in both groups	[70]
ABILITY	Biodegradable polymer sirolimus-eluting stent vs. durable polymer everolimus-eluting stent	131 patients with diabetes and coronary artery disease	Neointimal volume at 9–12-month follow-up Target lesion failure	29.11 ± 18.90 mm^3^ vs. 25.48 ± 17.04 mm^3^; *p* = 0.40 Target lesion failure: 21.2% vs. 19.6%	[71]
BIOHEART-II	Bioresorbable sirolimus-eluting stent vs. cobalt–chromium everolimus-eluting stent	434 patients with coronary artery disease	12-month in-segment late loss 12-month proportion of covered struts assessed on optical coherence tomography Target lesion failure at 3 years	12-month in-segment late loss 0.17 ± 0.38 mm vs. 0.14 ± 0.24 mm; p_non-inferiority_ < 0.001 The proportion of covered struts was 97.9% vs. 98.5%; p_non-inferiority_ < 0.001; p_superiority_ = 0.91 Target lesion failure at 3 years: 5.6% vs. 5.2%; *p* = 0.84	[72]
TARGET All Comers Trial	Biodegradable polymer sirolimus-eluting stent vs. durable polymer everolimus-eluting stent	1653 patients with acute or chronic coronary syndrome	Target lesion failure, ischemia-driven target revascularization and definite or probable ST in both groups at 5 years	Acute coronary syndrome: target lesion failure 16.0% vs. 14.9%; *p* = 0.70, ischemia-driven target lesion revascularization 5.6% vs. 8.3%; *p* = 0.17, and definite/probable ST 2.7% vs. 4.6%; *p* = 0.18 Target lesion failure 18.0% vs. 17.4%; *p* = 0.82, ischemia-driven target lesion revascularization 6.4% vs. 5.0%; *p* = 0.37, and definite/probable ST 3.0% vs. 1.8%; *p* = 0.26	[73]
DUTCH PEERS	Cobalt–chromium zotarolimus-eluting stent vs. platinum–chromium everolimus-eluting stent	1811 patients aged 18 years and older who required a percutaneous coronary intervention with implantation of DES	Target-vessel failure—a composite of safety (cardiac death or target-vessel-related myocardial infarction) and efficacy (target-vessel revascularization) at 12 months	Target-vessel failure: 55 patients (6%) vs. 47 patients (5%); p_non-inferiority_ = 0.006 Definite ST: 3 (0.3%) patients vs. 6 (0.7%) patients; *p* = 0.34	[74]
RESOLUTE	Zotarolimus-eluting stent vs. everolimus-eluting stent	2292 adult patients with chronic, stable coronary artery disease or acute coronary syndrome	Patient-oriented composite endpoint (combination of all-cause mortality, myocardial infarction, and any revascularizations) at 5-year follow-up Device-oriented composite endpoint (combination of cardiac death, target vessel myocardial infarction, and clinically indicated target lesion revascularization) at 5-year follow-up Major adverse cardiac events (combination of all-cause death, all myocardial infarction, emergent coronary bypass surgery, or clinically indicated target lesion revascularization) at 5-year follow-up	Patient-oriented composite endpoint 35.3% vs. 32.0%; *p* = 0.11 Device-oriented composite endpoint 17.0% vs. 16.2%; *p* = 0.61 Major adverse cardiac events 21.9% vs. 21.6%; *p* = 0.88 definite/probable ST 2.8% vs. 1.8%; *p* = 0.12	[37]
SORT-OUT III	Zotarolimus-eluting stent vs. sirolimus-eluting stent	2332 adult patients with chronic stable coronary artery disease or acute coronary syndrome and at least one target lesion	Primary endpoint (a composite of major adverse cardiac events within 9 months: cardiac death, myocardial infarction, and target vessel revascularization) Intention-to-treat analyses were done at 9-month and 18-month follow-up	Primary endpoint at 9-month follow-up: 72 (6%) vs. 34 (3%); *p* < 0.001 Primary endpoint at 18-month follow-up: 113 (10%) vs. 53 (5%); *p* < 0.001 All-cause-mortality at 9-month follow-up: 25 (2%) vs. 18 (2%); *p* = 0.28 All-cause mortality at 18-month follow-up: 51 (4%) vs. 32 (3%); *p* = 0.035	[75]

## Abbreviations

The following abbreviations are used in this manuscript:

CAD	Coronary artery disease
ACS	Acute coronary syndrome
NSTEMI	Non-ST elevation myocardial infarction
STEMI	Myocardial infarction with ST elevation
PCI	Percutaneous coronary intervention
CABG	Coronary artery bypass grafting
POBA	Plain old balloon angioplasty
BMS	Bare metal stent
ISR	In-stent restenosis
DES	Drug-eluting stent
ST	Stent thrombosis
mTOR	Mammalian target of rapamycin
DNA	Deoxyribonucleic acid
DCB	Drug-coated balloon
BRS	Bioresorbable scaffold system
GES	Gene-eluting stent
BVS	Bioabsorbable vascular stent
